# QSPR analysis of some agonists and antagonists of α-adrenergic receptors

**DOI:** 10.1007/s00044-014-1130-x

**Published:** 2014-07-15

**Authors:** Piotr Kawczak, Leszek Bober, Tomasz Bączek

**Affiliations:** 1 Department of Pharmaceutical Chemistry, Medical University of Gdansk, 80-416 Gdańsk, Poland; 2 POLPHARMA SA Pharmaceutical Works, 83-200 Starogard Gdański, Poland; 3 Division of Human Anatomy and Physiology, Institute of Health Sciences, Pomeranian University of Słupsk, 76-200 Słupsk, Poland

**Keywords:** Adrenergic receptors, Agonists, Antagonists, PCA, FA, RP-LC

## Abstract

Thirty-three compounds belonging to the sympatholytics and sympathomimetics were analyzed during the study. The biological activity data for the parameters of binding affinity to the α_1_- and α_2_-adrenergic receptors together with parameters of the logarithm of the partition coefficient *n*-octanol/water (log *P*) were performed using a semi-empirical calculations methods for isolated molecules (in vacuo) and for the molecules placed in an aqueous environment. Additionally, the chromatographic retention data were used as extra dependent variables of the structural parameters for a part of the considered compounds. Finally, all those groups of parameters were analyzed using MLR, PCA, and FA methods for the classification of studied compounds according to their chemical structures and pharmacological activity to the adrenoceptors.

**Electronic supplementary material:**

The online version of this article (doi:10.1007/s00044-014-1130-x) contains supplementary material, which is available to authorized users.

## Introduction

Stimulants of α_1_- and α_2_-adrenergic receptors belong to the sympathomimetics stimulating sympathetic autonomic nervous system. Depending on the receptor that is stimulated, various physiological effects such as contractions of vascular smooth muscle, spasm of sphincter, mydriasis, etc. are observed (Schmitz *et al.*, 1981; Robinson and Hudson, 1998; Fitzpatrick *et al.*, 2004).

Sympathomimetic natural neurotransmitter, noradrenaline, resulting from the amino acid—tyrosine. Because noradrenaline is an unstable compound (which is prone to oxidation) and further is pointless cause all of the physiological effects for which noradrenaline is responsible. Man has developed a number of synthetic stimulants which have a selective effect with respect to the physiological receptor α_1_ and α_2_, in particular postsynaptic. On the other hand, the agents that block α_1_ and α_2_-adrenergic receptors (selectively or not) belong to the sympatholytics (adrenolytics), i.e., agents inhibiting the sympathetic nervous system: imidazoline derivatives (phentolamine, tolazoline) block both types of α receptors, derivatives of piperazinchinazolin (prazosin, doxazosin, terazosin) block selectively α_1_ receptors, ergot alkaloids block predominantly α_2_ receptors, and yohimbine blocks selectively α_2_ receptors. Blocking agents of α-adrenergic receptors are most commonly used as cardiovascular drugs: α_1_-blockers as antihypertensive drugs, α_2_-blockers as hypertensive ones; ergot alkaloids have a contractive effect on the uterus, but their hydrogenated derivatives are devoid of this activity, improving peripheral blood. Non-specific α-blockers accelerate the heart rate, dilate peripheral vessels, increasing the contractility of intestines and secretory activity of gastric mucosal (Schmitz *et al.*, 1981; Robinson and Hudson, 1998; Fitzpatrick *et al.*, 2004).

Over time, agonists and antagonists of adrenoceptors have become the subject of a number of works in the field of molecular modeling, lipophilicity, and structure–activity as well as 3D QSAR (Eric *et al.*, 2004; Balogh *et al.*, 2007, 2009; Nikolic *et al.*, 2008; Zhao *et al.*, 2011; Yadav *et al.*, 2013).

Timmermans and co-workers have published interesting series of papers about agonists and antagonists of adrenoceptors in order to characterization and classification of selected molecules (Timmermans *et al.*, 1981, 1984; Timmermans and Van Zwieten, 1982). In one of these papers (Timmermans *et al.*, 1984), the authors have considered hypotensive and hypertensive activity relationships of α-adrenomimetics and experimentally determined logarithm of the *n*-octanol/water partition coefficient, log *P*, and also experimentally determined binding affinity to α_1_ and α_2_ receptors. Obtained by the authors, relationships according to the activity and logarithm of the partition coefficient were unsatisfactory. More preferably shown themselves to be the relationships in term of binding affinity (*R* > 0.9). For α-adrenolytics, authors presented relationships according to indexes of α_1_/α_2_ adrenoceptor antagonist selectivity in vivo and indexes of α_1_/α_2_ adrenoceptor antagonist of pre and postsynaptic selectivity in vivo considering selectivity indexes of binding of α_1_/α_2_ adrenoreceptor to the corresponding ones (*R* > 0.9).

The objective of the presented study was to analyze the biological activity data (Timmermans *et al.*, 1984), the parameters of binding affinity to the α_1_ and α_2_ receptors together with parameters of the logarithm of the partition coefficient *n*-octanol/water (log *P*) using semi-empirical calculations methods (Bączek, 2006; Bodzioch *et al.*, 2010) for isolated molecules (in vacuo) and the for the molecules placed in an aqueous environment. In addition, for a part of the consideration, compounds were available chromatographic retention data (Nasal *et al.*, 1997), which were used as the dependent variables of the structural parameters.

The aim of this study was to demonstrate the characteristics of both common and differentiating the analyzed compounds in terms of physicochemical and pharmacological effects.

## Experimental procedure

### Molecules

**Fig. 1:**
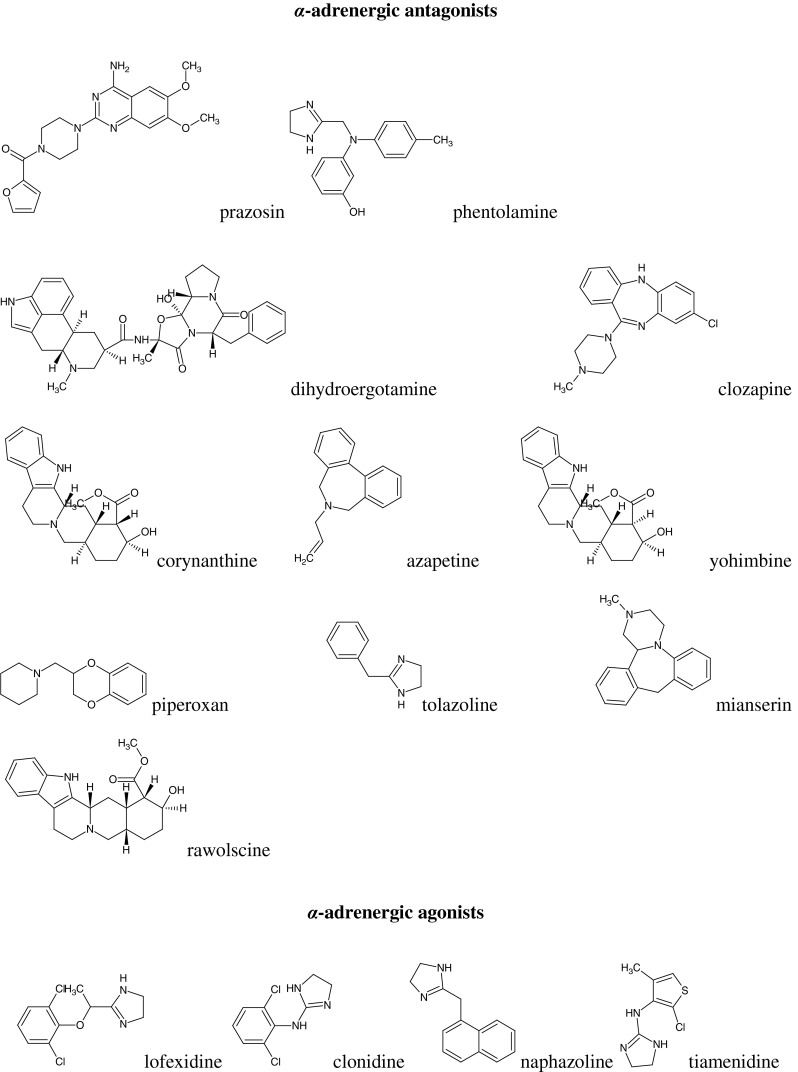

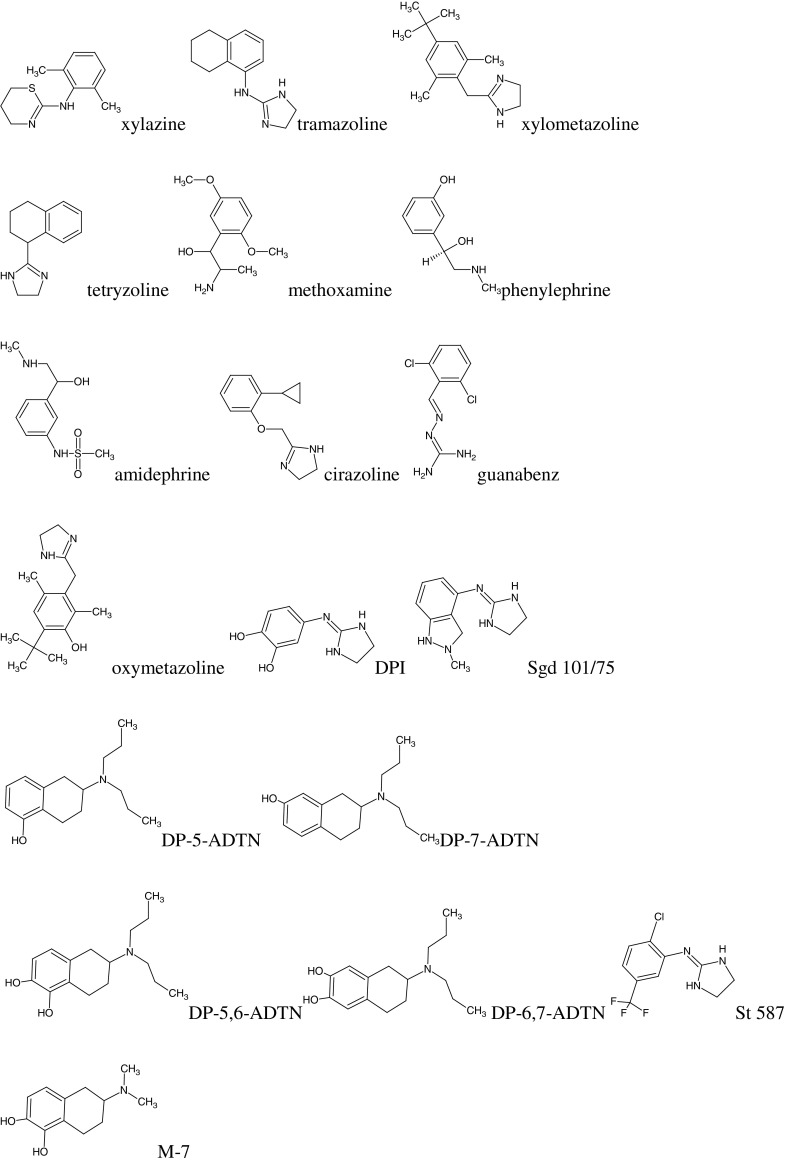
Structural formulas of compounds studied

The following compounds were selected for testing according to reference (Timmermans *et al.*, 1984):

- α-adrenergic antagonists (AN): prazosin, phentolamine, dihydroergotamine, clozapine, corynanthine, azapetine, yohimbine, piperoxan, tolazoline, mianserin, rauwolscine;
- α-adrenergic agonists (AG): lofexidine, clonidine, naphazoline, tiamenidine, xylazine, tramazoline, xylometazoline, tetryzoline, methoxamine, phenylephrine, amidephrine, cirazoline, guanabenz, oxymetazoline, and eight compounds of an experimental structures, marked as symbols: DPI, Sgd 101/75, DP-5-ADTN, DP-7-ADTN, DP-5,6-ADTN, DP-6,7-ADTN, St 587, and M-7 (Fig. 1).

### Biological activity data

The study used the literature-quoted data of biological activity (Timmermans *et al.*, 1984), are presented in Table 1S. The activity of α-adrenergic agonists—antihypertensive activity was derived from the stimulation of central α_2_-adrenoceptors, pC_25_. The authors expressed data for pC_25_ in μmol/kg. The values of pC_25_ were available for lofexidine, clonidine, naphazoline, tiamenidine, xylazine, tramazoline, xylometazoline, and tetryzoline.

For the α-adrenergic, antagonists were used:

- antagonistic activity against phenylephrine induced via α_1_-adrenoceptors vasoconstriction in rats, pA_2 post_ (α_1_)—in vivo,
- antagonistic activity of phenylephrine- or norepinephrine-induced stenosis of isolated rabbit pulmonary artery through α_1_-adrenereceptors post, pA_2__post_ (α_1_)—in vitro.

Activities expressed as pA_2_ were derived from the equation (Timmermans *et al.*, 1984):

1 $${\text{pA}}_{2} = { \log }\left( {{\text{dose}}\;{\text{ratio}} - 1} \right) - { \log }({\text{antagonist}}\;{\text{concentration}})$$

### Chromatographic and lipophilicity data

The values of the logarithm of partition coefficient, log *P*, were derived from the paper by Timmermans *et al.* (1984), and they are refer to compounds: lofexidine, clonidine, naphazoline, tiamenidine, xylazine, tramazoline, xylometazoline, tetryzoline, cirazoline, St-587, and oxymetazoline (Table 2S).

Chromatographic data were derived from the article by Nasal *et al.* (1997), and they are refer to compounds: lofexidine, clonidine, naphazoline, tiamenidine, xylometazoline, tetryzoline, cirazoline, oxymetazoline, prazosin, phentolamine, and tolazoline (Table 2S). These are the values of the logarithms of retention factors determined on Chiral AGP (log *k*_AGP_), immobilized artificial membranes IAM.PC.MG (log *K*_IAM_) and also the logarithm values of lipophilicity coefficients determined by the policratic method on Suplex pKb-100, pH 7.4 (log *k*_w7.4Su_), Spheri RP-18, pH 2.5 (log *k*_w2.5Sp_), and Aluspher RP select B, pH 7.3 (log *k*_w7.3Al_).

### Molecular descriptors

The non-empirical structural indicators, i.e., quantum-chemical indicators, were calculated in the study. The PCM (Polarizable Continuum Model) method (Tomasi and Persico, 1994; Tomasi *et al.*, 2005; Caricato and Scalmani, 2011) would be prefer in the ab initio calculations for the all tested compounds as we previously presented (Bober *et al.*, 2012a, b), but the size of some analyzed molecules (e.g., alkaloids of α-adrenergic antagonists with the number of atoms above 50) complicated or even prevented the use of ab initio methods under these consideration on a standard class PC. The only choice was to use a semi-empirical method for the whole group of analyzed compounds by placing one by one molecule in the environment of water molecules. The structure of the tested compounds was studied by molecular modeling using HyperChem Release 8.0 (Hypercube Inc., Gainesville, FL, USA) software. The geometry of the molecule was initially optimized by molecular mechanics MM+ and then using the semiempirical method RM1 (HyperChem^®^ Computational Chemistry, 1996). After completing the optimization a single point calculation was performed. The molecule was placed in a periodic box, which dimensions was selected in such a way that program has placed within around 40 water molecules, and the optimization of the geometry was repeated in an environment of water molecules by RM1. Among the quantum-chemical indices were considered: total energy (TE), binding energy (BE), electron energy (EE), heat of formation (HF) energy, highest occupied molecular orbital (E_HOMO), the energy of the lowest unoccupied molecular orbital (E_LUMO), and the difference between HOMO and LUMO energy defined as the energy gap (EG). Moreover, the following values were used: the largest positive charge on the electron atoms (MAX_POS), the largest negative charge on the electron atoms (MAX_NEG), the difference between the largest positive and negative charge (DELTA_Q), the total dipole moment (TDM), the mean polarizability (MPOL), and energy values for the most long-term transition of electron EL (for which a power oscillator >0). Values of TE expressed in atomic energy units a.u. or Hartree (1 Hartree = 2625.552 kJ mol^−1^, or 627.552 kcal mol^−1^ or 27.2116 eV), energies of HOMO, LUMO, and gap energy expressed in eV (counted above values of a.u. to eV), electron spatial extent in eBohr^−3^ (Bohr = 0.5292 × 10^−10^ m = 0.5292 Å). The values of electron density and electron charges on the atoms are in units of elementary charge (${e^-}$), the dipole moment is expressed in Debye (D), and the average polarizability in Bohr^−3^ (Bohr = 0.5292 × 10^−10^ m = 0.5292 Å). Using QSAR module (QSAR Properties Module) of HyperChem 8.0 other non-empirical parameters were calculated—parameter values dependent on the geometry of the molecule: the surface of the molecule accessible to the solvent (SA), expressed in Å^2^, the volume of the particle (*V*) in Å^3^, and the hydration energy (HE), in kcal mol^−1^ for both type of structures optimized by RM1 in vacuo and in the surrounding water molecules.

However, the smaller size structure of α-adrenergic agonists was additionally studied using the molecular modeling software Gaussian 03 W (v03, Gaussian Inc., Wallingford, CT, USA). The geometry of the molecules was optimized using Hartree–Fock restricted 6-31G (*d*, *p*) also known as 6-31G** (http://www.gaussian.com/). The quantum-chemical indices considered from that calculations were as follows: electronic spatial extent (ESE)—defined as the area including the volume around the particles beyond which the electron density is less than 0.001 eBohr^−3^ describing the sensitivity of the molecule to the electric field, TE, EHOMO, ELUMO, EG, MAX_POS, MAX_NEG, DELTA_Q, TDM, and finally the isotropic polarizability (IPOL) expressed in eBohr^−3^.

### Statistical analysis

The retention data and the data of biological activity of the compounds studied were related to their structural indicators under stepwise, progressive, and multiparametric regression analysis (multiple regression) and calculated with the use of Statistica 10 (v10, StatSoft, Tulsa, OK, USA, 2011) installed on a personal computer. As a preliminary principal component analysis (PCA) and factor analysis (FA) were performed to make the initial classification of compounds under the consideration.

## Results and discussion

The numerical values of 16 structural parameters derived from the quantum-chemical calculations in vacuo for all 33 considered compounds are shown in Table 3S and derived from the quantum-chemical calculations in the aquatic environment for all 33 considered compounds are presented in Table 4S. The numerical values of the 10 structural parameters derived from quantum-chemical calculations in vacuo for 22 considered compounds (α-adrenergic agonists) obtained by the PCM (Polarizable Continuum Model) method are shown in Table 5S.

After the PCA and FA for a set of in vacuo calculations found that the greatest impact on the first factor had the mean polarizability (MPOL) and the molecular volume of the particle (*V*), followed by particle surface area (SA), EE, BE, and finally TE and HF. Additionally, it was confirmed by cross-validation method that the above-mentioned three types of energy (TE, BE, EE) are correlated. The second factor was clearly influenced by the difference between the largest positive and negative charge (Δ*Q*), the largest positive charge on the atom (MAX_POS) and the largest negative charge on the atom (MAX_NEG), followed by the energy of the lowest unoccupied molecular orbital (E_LUMO).

**Fig. 2:**
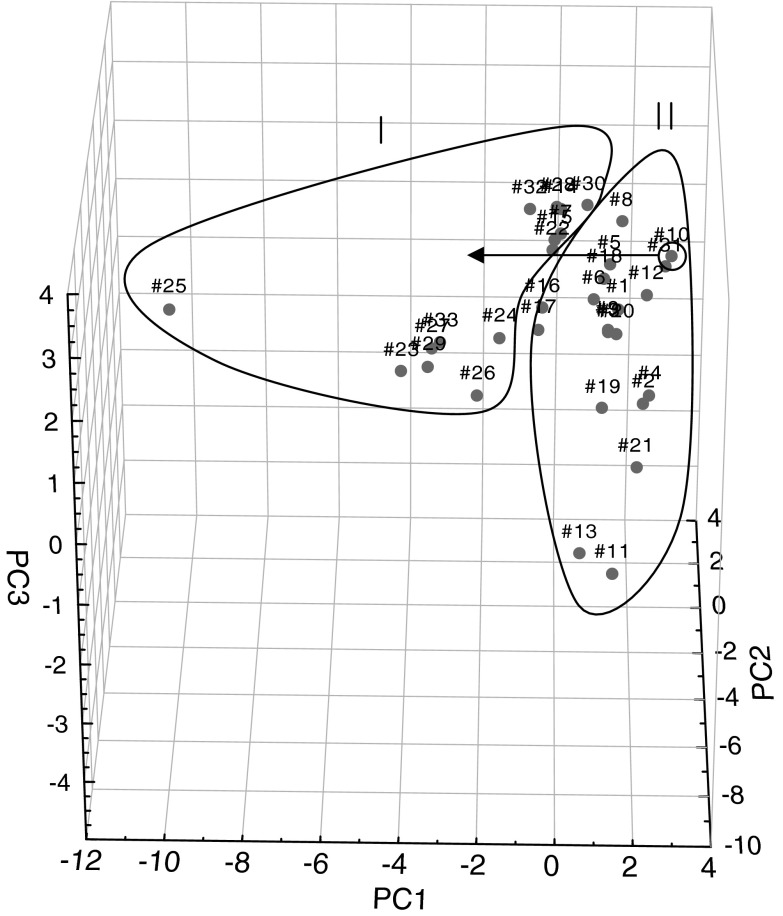
Three-dimensional scatter plots of the loadings of the first three factors (PC1—42,74 %, PC2—24,47 %, PC3—12,16 %) obtained by PCA of structural parameters derived from the quantum-chemical calculations in vacuo for all 33 considered compounds; where: I—α-adrenergic antagonists (AN) and II—α-adrenergic agonists (AG)

**Fig. 3:**
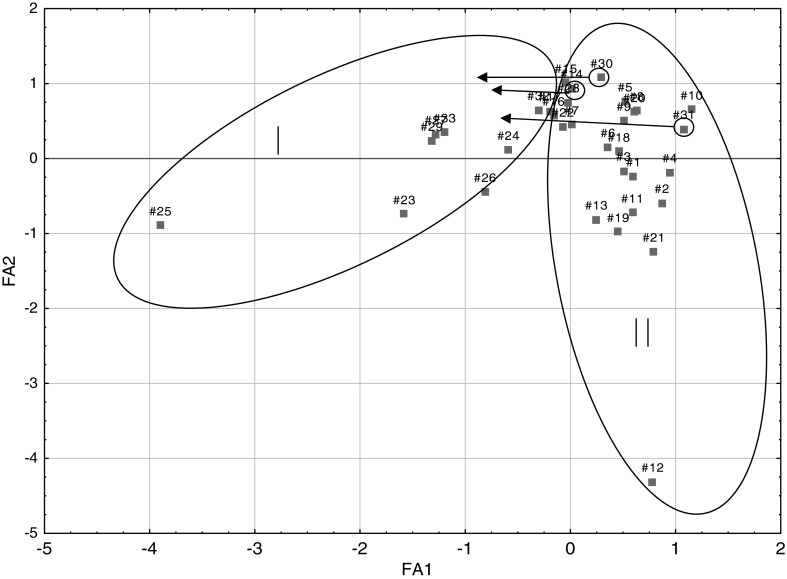
Two-dimensional scatter plots of the loadings of the first two factors (FA1—42,74 %, FA2—24,47 %) obtained by FA of structural parameters derived from the quantum-chemical calculations in vacuo for all 33 considered compounds; where I—α-adrenergic antagonists (AN) and II—α-adrenergic agonists (AG)

**Fig. 4:**
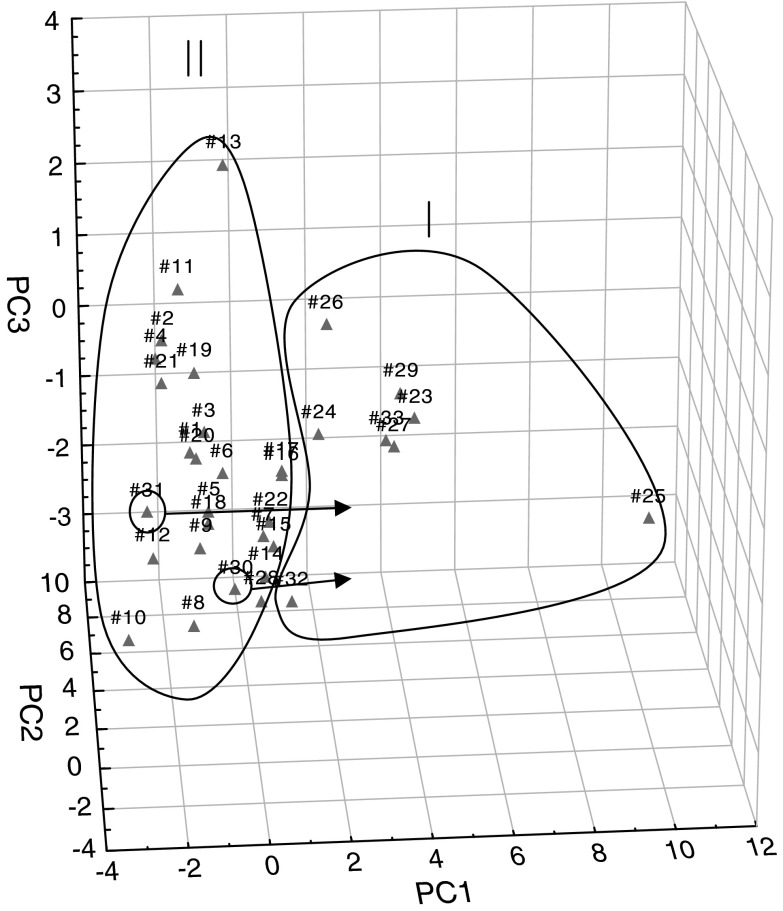
Three-dimensional scatter plots of the loadings of the first three factors (PC1—42,59 %, PC2—25,49 %, PC3—10,90 %) obtained by PCA of structural parameters derived from the quantum-chemical calculations in the aquatic environment for all 33 considered compounds; where I—α-adrenergic antagonists (AN) and II—α-adrenergic agonists (AG)

**Fig. 5:**
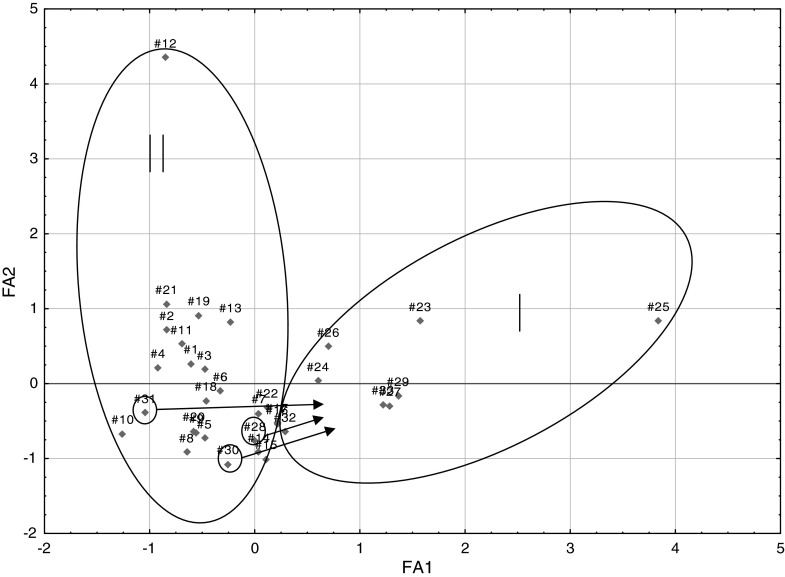
Two-dimensional scatter plots of the loadings of the first two factors (FA1—42,59 %, FA2—25,49 %) obtained by FA of structural parameters derived from the quantum-chemical calculations in the aquatic environment for all 33 considered compounds; where I—α-adrenergic antagonists (AN) and II—α-adrenergic agonists (AG)

Comparing Figs. 2 and 3 from PCA and FA analyses, it can be seen that between the graphs of the distribution of points corresponding to individual cases relative to each other is very similar, if not identical. It is possible to extract two sets (clusters), including single points for α-adrenoceptor agonists (numbers of compounds 1–22)—II and α-adrenoceptor antagonists (23–33)—I. Furthermore, some of the antagonists: azapetine (28), piperoxane (30), tolazoline (31), and mianserine (32) are compounds having a non-specific effects on α-receptors, so it is possible that they may be partially mixed with a set of agonists, in presented work, they were marked with circle on Figs. 2, 3, 4, and 5 and pointed to the cluster with other antagonists.

In the next step, PCA and FA were performed for the same set of calculation in an aqueous medium. Comparing the obtained results, it was noted that the application of structural parameters calculated in terms of hydration has made no noticeable changes. Points corresponding to both variables and statistical cases were slightly shifted, however, the distribution of points unchanged and it was similar to the one presented in the discussion for the analysis of molecules calculated in vacuo (Figs. 4, 5).

It is difficult to determine whether the model based on the placing of the molecule in the present periodic box surrounded by water molecules with the creation of hydrogen bonds and the geometry optimization of the model is worse or better than the PCM, which consists in placing the particles presented in the environment, such as the dielectric constant of the solvent. On the other hand, using PCM model additional parameters are calculated characterizing the system, but also very important is a total number of cases that can be clearly presented.

The log *k*, chromatographic relationships for the structures of α-adrenergic agonists and some antagonists optimized in vacuo and in the aquatic environment as the results of muliregression analysis are presented in Table 1. For the structures of α-adrenergic agonists optimized in vacuo with the PCM method is presented in Table 2, and finally the activity relationships for the structures of α-adrenergic agonists and antagonists optimized in vacuo and in aquatic environment are presented in Table 3.

**Table 1:** The relationships for the structures of α-adrenergic agonists and some antagonists optimized in vacuo and in aquatic environment statistical parameters: *R*, *s*, *F* and *P* of regression equation log *k* = *k*_0_ + *k*_1_Descriptor_1_ + *k*_2_Descriptor_2_, where *n* = 11

*k*_1_Descriptor_1_	*k*_2_Descriptor_2_	*R*	*s*	*F*	*P*
*In vacuo*					
log *k*_AGP_					
0.9019 ± 0.1440 V	–	0.9019	0.1055	39.2375	0.0001
log *k*_IAM_					
−0.9418 ± 0.1121 BE	–	0.9418	0.1633	70.5851	0.0001
log *k*_w7.4Su_					
−0.9596 ± 0.0938 BE	–	0.9596	0.2424	104.5626	0.0001
log *k*_w2.5Sp_					
−1.6761 ± 0.1742 BE	1.0907 ± 0.1742 TE	0.9636	0.1634	51.8941	0.0001
*Hydrated*					
log *k*_AGP_					
0.9042 ± 0.1426 V	–	0.9042	0.1043	40.3182	0.0001
log *k*_IAM_					
−0.9418 ± 0.1121 BE	–	0.9418	0.1632	70.6113	0.0001
log *k*_w7.4Su_					
−1.0316 ± 0.0726 BE	0.02163 ± 0.0726 TDM	0.9811	0.1769	102.6939	0.0001
log *k*_w2.5Sp_					
−1.6752 ± 0.1740 BE	1.0896 ± 0.1740 TE	0.9636	0.1633	51.9731	0.0001

**Table 2:** The relationships for the structures of α-adrenergic agonists optimized in vacuo; by PCM method; statistical parameters: *R*, *s*, *F* and *P* of regression equation log *k*_(column)_ = *k*_0_ + *k*_1_Descriptor_1_, where *n* = 8

*k*_1_Descriptor_1_	*R*	*s*	*F*	*P*
log *k*_IAM_				
0.9420 ± 0.1371 IPOL	0.9420	0.1271	47.2322	0.0005
log *k*_w7.4Su_				
0.9714 ± 0.0968 ESE	0.9714	0.1499	100.6252	0.0001
log *k*_w2.5Sp_				
0.9527 ± 0.1240 IPOL	0.9527	0.1994	59.0060	0.0002
log *k*_w7.3Al_				
0.9295 ± 0.1505 ESE	0.9295	0.2286	38.1378	0.0008

**Table 3:** The activity relationships for the structures of α-adrenergic antagonists and agonists optimized in vacuo and in aquatic environment; statistical parameters: *R*, *s*, *F* and *P* of regression equation: pA_2_ (α_1_) _in vivo_/pA_2_ (α_1_) _in vitro_/pC_25_ = *k*_0_ + *k*_1_Descriptor_1_ + *k*_2_Descriptor_2_

*k*_1_Descriptor_1_	*k*_2_Descriptor_2_	*R*	*s*	*F*	*P*
pA_2_ (*α*_*1*_) _in vivo_, in vacuo, *n* = 11					
−0.6287 ± 0.1622 HE	−0.5189 ± 0.1622 E_LUMO	0.8935	0.4463	15.8397	0.0016
pA_2_ (*α*_*1*_) _in vitro_, in vacuo, *n* = 11					
−0.6398 ± 0.1674 E_LUMO	−0.4957 ± 0.1674 HE	0.8861	0.4808	14.6273	0.0021
pA_2_ (*α*_*1*_) _in vivo_, hydrated, *n* = 11					
−0.6089 ± 0.1553 HE	−0.5558 ± 0.1553 E_LUMO	0.9026	0.4279	17.5874	0.0012
pA_2_ (*α*_*1*_) _in vitro_, hydrated, *n* = 11					
−0.8639 ± 0.1575 E_LUMO	0.4811 ± 0.1575 HF	0.8998	0.4526	17.0163	0.0013
pC_25_, in vacuo, *n* = 8					
−0.8672 ± 0.2033 E_LUMO	–	0.8672	0.4310	18.1891	0.0053
pC_25_, hydrated, *n* = 8					
−0.8798 ± 0.1941 E_LUMO	–	0.8798	0.4114	20.5463	0.0040

According on the chromatographic relationships for the structures of α-adrenergic agonists and some antagonists optimized in vacuo, they are characterized by the values of the regression coefficients *R* > 0.9. Relatively strong dependencies were obtained also for the columns serving as a models of biological systems (IAM, AGP), *R* ~ 0.94 and ~0.9, respectively. Among the analyzed independent variables the most frequent appears BE and as the further parameter TE for the Spheri column, *R* ~ 0.96. Additionally, for the AGP, molecular volume (*V*) appears in column.

Between the chromatographic relationships for the structures of α-adrenergic agonists and some antagonists optimized in aquatic environment, similar dependencies were observed. Furthermore, for the Suplex column, a second parameter appears the TDM with *R* ~ 0.98.

On the other hand, analyzing the relationships for the structures of only α-adrenergic agonists, *n* = 8, optimized in vacuo by PCM method in all cases the values of the regression coefficients *R* > 0.93 with only one independent variable. The most frequent parameter appeared isotropic polarizability (IPOL), *R* ~ 0.94 for the IAM column and *R* ~ 0.95 for the Spheri column. However, for the Suplex and Aluspher columns appeared electronic spatial extent (ESE), with *R* ~ 0.97 and ~0.93, respectively.

Analyzing the dependencies of α-adrenergic agonists and log *P* two independent variables appeared only as a statistically significant parameters in vacuo: MAX_NEG and TDM, with the regression coefficient, *R* ~ 0.9, that could demonstrate the importance of bulkiness type parameters and associated dispersion interactions, to a lesser extent polar parameters.

For the antihypertensive activity of agonists (pC_25_) and a relatively not too large number of cases (*n* = 8), relationship with only one the independent variable—the lowest energy unoccupied molecular orbital (E_LUMO) with a regression coefficient value *R* ~ 0.87 in vacuo and *R* ~ 0.88 in the case of hydrated structures—was obtained.

For the biological activity of antagonists (*n* = 11), statistically significant dependencies of pA_2_ (α_1_) in vivo and in vitro activity for the both hydrated and non-hydrated molecules were obtained. In the case of in vacuo structures with pA_2_ (α_1_)_in vivo_ as the first parameter appears HE and as the second the lowest energy unoccupied molecular orbital (E_LUMO), with *R* ~ 0.89, while with pA_2_ (α_1_)_in vitro_ there is a the inverse order of the same parameters also with *R* ~ 0.89. On the other hand, In the case of hydrated structures with pA_2_ (α_1_)_in vivo_ as the first parameter appears again HE and as the second the lowest energy unoccupied molecular orbital (E_LUMO), with *R* ~ 0.90, whereas with pA_2_ (α_1_)_in vitro_ there is an inverse order of the same parameters also with *R* ~ 0.89, whereas as the first parameter appears the lowest energy unoccupied molecular orbital (E_LUMO) and as the second the HF, with *R* ~ 0.90.

It can be concluded that for the parameters of the binding affinity of the receptor, a major role is played by E_LUMO energy orbitals, which may indicate the nature of the interactions between the drug molecule and receptor.

It seems that the regression is mostly affected by the type of the dependent variable, and in fact the complexity of the phenomena affecting the measured value of this variable, as well as the uncertainty of measurement of the variable. The dependent variables—physicochemical parameters (such as chromatographic retention factors and hydrophobicity coefficients) probably have a smaller number of phenomena which influence its value than the biological parameters, and the uncertainty of their measurement seems to be smaller. The parameters characterizing the biological activity (authors assigned them in living organisms or living tissues) are more complex nature than the phenomenon of chromatographic retention processes, so often they may possess not so preferred statistical characteristics (i.e., accuracy and precision), which all results in a lower value of R.

## Concluding remarks

Based on the above discussion the following conclusions can be drawn.

Out of the considered 16 molecular parameters (quantum-chemical and QSAR), the greatest impact on the spatial distribution (and classification) have the average polarizability and molecular volume, followed by particle surface area and three type of energies electron, binding and total. On the other hand, it appears with smallest impact: the difference between the largest positive and negative charge, the largest positive charge on the atom, and the largest negative charge on the atom.

The greatest impact on the values of chromatographic retention has BE and sometimes TE or TDM; instead of PCM method it informs us about equally important influence of isotropic polarizability and electronic spatial extent.

Between the relationships together with the chromatographic parameters appear high values of the regression coefficient (*R* > 0.95), sometimes even with one independent variable—BE, which assumes the existence of dependency of a function type.

Not found, the significant effect of hydration (the calculation method for the structure of hydrated “periodic box”) for the statistical analysis (PCA, FA and MLR) in comparison to the results of the analysis for the structure optimized in vacuo.

Analyzing the relationships resulting from the correlation with parameters of biological activity, the most frequent dependence is that with the value of lowest energy unoccupied molecular orbital probably playing a crucial role as a result of the agonistic and antagonistic activity on the α-adrenergic receptors. It seems to converge with the results on similar structures and effect on adrenoceptors (Eric *et al.*, 2004; Nikolic *et al.*, 2008) suggesting the meaning of HOMO energy orbitals. The importance of LUMO and HOMO energy orbitals for analyzed parameters characterizing the biological activity to α_1_ and α_2_ receptors indicates the importance of the electron-donor–acceptor interaction within the drug–receptor interactions.

## Electronic supplementary material

Below is the link to the electronic supplementary material.

Supplementary material 1 (DOC 235 kb)
