# Supplementary material for: QSPR analysis of some agonists and antagonists of α-adrenergic receptors
Source: Med Chem Res. 2014 Jul 15;24(1):372–82. doi: 10.1007/s00044-014-1130-x (PMC4284397; doi:10.1007/s00044-014-1130-x)
Supplement: Supplementary file 1 — Supplementary material 1 (DOC 235 kb) [file 44_2014_1130_MOESM1_ESM.doc]

Supplementary files

**Table 1S.** The activity values of pC_25_ for *α*-adrenergic agonists and the values of pA_2_ (*α_1_*) *in vivo* and pA_2_ (*α_1_*) *in vitro* for *α*-adrenergic antagonists according to Ref. (Timmermans *et al.*, 1984).

| **Compound** | **pC_25_** | **pA_2_ (*α_1_*)** *in vivo* | **pA_2_ (*α_1_*)** *in vitro* |
| --- | --- | --- | --- |
| **lofexidine** | 2.09 | - | - |
| **clonidine** | 2.04 | - | - |
| **naphazoline** | 0.95 | - | - |
| **tiamenidine** | 0.69 | - | - |
| **xylazine** | 0.62 | - | - |
| **tramazoline** | 0.55 | - | - |
| **xylometazoline** | 0.26 | - | - |
| **tetryzoline** | -0.16 | - | - |
| **prazosin** | - | 8.33 | 8.70 |
| **phentoloamine** | - | 6.95 | 7.49 |
| **dihydroergotamine** | - | 6.68 | 7.55 |
| **clozapine** | - | 6.29 | 7.43 |
| **corynanthine** | - | 6.25 | 6.60 |
| **azapetine** | - | 6.09 | 6.96 |
| **yohimbine** | - | 5.72 | 6.40 |
| **piperoxane** | - | 5.71 | 6.06 |
| **tolazoline** | - | 5.66 | 5.41 |
| **mianserin** | - | 5.41 | 6.60 |
| **rauwolscine** | - | 5.12 | 5.89 |

**Table 2S.** The values of chromatographic parameters for some of *α*-adrenergic agonists and antagonists, according to Ref. (Nasal *et al.*, 1997) together with the values of log *P* of *α*-adrenergic agonists according to Ref. (Timmermans *et al.*, 1984).

| **Compound** | **log *k_AGP_*** | **log *k_IAM_*** | **log *k_w7.4Su_*** | **log *k_w2.5Sp_*** | **log k_w7.3Al_** | **log *P*** |
| --- | --- | --- | --- | --- | --- | --- |
| **lofexidine** | 0.965 | 0.879 | 1.479 | 0.509 | 1.581 | 0.73 |
| **clonidine** | 0.847 | 0.410 | 1.138 | 0.201 | 1.163 | 0.85 |
| **naphazoline** | 1.092 | 0.895 | 1.297 | 0.678 | 1.706 | -0.52 |
| **tiamenidine** | 0.808 | 0.434 | 0.834 | 0.308 | 1.000 | -0.17 |
| **xylometazoline** | 1.158 | 1.362 | 2.380 | 1.920 | 2.475 | 0.40 |
| **tetryzoline** | 0.822 | 0.553 | 0.917 | 0.671 | 1.001 | -0.90 |
| **cirazoline** | 1.082 | 0.940 | 1.374 | 0.693 | 1.948 | 0.53 |
| **oxymetazoline** | 1.108 | 1.216 | 2.312 | 1.578 | 2.319 | -0.32 |
| **prazosin** | 1.390 | 1.594 | 2.948 | 0.909 | 1.442 | - |
| **phentoloamine** | 1.264 | 1.340 | 1.970 | 1.289 | 2.386 | - |
| **tolazoline** | 0.586 | 0.155 | 0.100 | 0.404 | 0.580 | - |
| **xylazine** | - | - | - | - | - | 1.34 |
| **tramazoline** | - | - | - | - | - | -0.62 |
| **St-587** | - | - | - | - | - | 1.54 |

**Table 3S.** The numerical values of 16 structural parameters derived from the quantum-chemical calculations *in* *vacuo* for all 33 considered compounds.

| **No.** | **Compound** | **TE** | **BE** | **EE** | **HF** | **E_HOMO** | **E_LUMO** | **EG** | **MAX_POS** | **MAX_NEG** | **DELTA_Q** | **TDM** | **MPOL** | **EL** | **SA** | **V** | **HE** |
| --- | --- | --- | --- | --- | --- | --- | --- | --- | --- | --- | --- | --- | --- | --- | --- | --- | --- |
| 1 | **lofexidine (AG)** | -112.24 | -4.522 | -657.87 | 0.483 | -9.0662 | -0.3377 | 8.7285 | 0.2550 | -0.4989 | 0.7538 | 4.039 | 117.99 | 3.836 | 421.87 | 690.42 | -2.30 |
| 2 | **clonidine (AG)** | -97.51 | -4.112 | -505.95 | -7.642 | -8.7400 | -0.4840 | 8.2560 | 0.4159 | -0.4878 | 0.9031 | 3.808 | 114.46 | 3.742 | 388.05 | 612.67 | -5.28 |
| 3 | **naphazoline (AG)** | -87.29 | -4.393 | -553.64 | 25.640 | -8.8253 | -0.4465 | 8.3788 | 0.2455 | -0.4985 | 0.7440 | 3.047 | 132.63 | 3.684 | 424.78 | 673.49 | -3.42 |
| 4 | **tiamenidine (AG)** | -86.24 | -3.492 | -456.15 | 5.692 | -8.4514 | 0.0794 | 8.5308 | 0.4789 | -0.4929 | 0.9718 | 3.593 | 107.81 | 3.573 | 382.25 | 600.64 | -5.61 |
| 5 | **xylazine (AG)** | -86.07 | -4.165 | -551.79 | 24.430 | -8.6676 | 0.3100 | 8.9776 | 0.2654 | -0.4141 | 0.6795 | 1.768 | 132.43 | 3.692 | 421.08 | 687.34 | -1.80 |
| 6 | **tramazoline (AG)** | -91.98 | -4.823 | -598.18 | 18.207 | -8.5534 | 0.2930 | 8.8464 | 0.4139 | -0.4978 | 0.9117 | 3.015 | 130.99 | 3.906 | 436.72 | 697.37 | -3.49 |
| 7 | **xylometazoline (AG)** | -101.81 | -5.844 | -753.02 | 23.560 | -8.7843 | 0.2756 | 9.0599 | 0.2580 | -0.4964 | 0.7544 | 3.021 | 142.48 | 3.794 | 486.26 | 818.44 | 0.77 |
| 8 | **tetryzoline (AG)** | -83.75 | -4.452 | -541.12 | 21.129 | -8.8185 | 0.5327 | 9.3512 | 0.2514 | -0.5146 | 0.7660 | 2.570 | 115.78 | 3.887 | 412.65 | 658.56 | -1.96 |
| 9 | **methoxamine(AG)** | -102.23 | -3.986 | -624.00 | 24.097 | -8.4054 | 0.5229 | 8.9283 | 0.1988 | -0.4873 | 0.6861 | 2.852 | 110.34 | 3.668 | 414.72 | 670.33 | -6.09 |
| 10 | **phenylephrine (AG)** | -79.29 | -3.217 | -410.37 | 18.581 | -9.0901 | 0.2191 | 9.3092 | 0.2188 | -0.4477 | 0.6665 | 0.193 | 88.35 | 3.774 | 370.09 | 567.03 | -13.80 |
| 11 | **DPI (AG)** | -93.62 | -3.508 | -493.39 | 15.957 | -7.7619 | 0.5130 | 8.2749 | 0.5011 | -0.5196 | 1.0207 | 5.971 | 120.13 | 3.683 | 378.03 | 585.23 | -20.46 |
| 12 | **amidephrine (AG)** | -111.75 | -3.873 | -669.72 | 25.293 | -9.3049 | -0.7692 | 8.5357 | 2.9671 | -0.9923 | 3.9595 | 4.962 | 116.79 | 3.831 | 449.70 | 717.22 | -11.00 |
| 13 | **Sgd 101/75(AG)** | -95.96 | -4.482 | -587.96 | 13.436 | -7.7961 | 0.0822 | 7.8783 | 0.4609 | -0.5001 | 0.9609 | 6.491 | 137.84 | 3.673 | 427.65 | 670.66 | -10.34 |
| 14 | **DP-5-ADTN (AG)** | -106.27 | -5.700 | -793.14 | 27.421 | -8.9154 | 0.5784 | 9.4938 | 0.2156 | -0.4059 | 0.6215 | 2.615 | 137.77 | 3.775 | 487.15 | 820.33 | -3.72 |
| 15 | **DP-7-ADTN (AG)** | -106.27 | -5.702 | -785.54 | 27.383 | -8.6139 | 0.5686 | 9.1825 | 0.2160 | -0.4056 | 0.6217 | 0.949 | 139.31 | 3.711 | 492.62 | 823.95 | -4.51 |
| 16 | **DP-5,6-ADTN (AG)** | -117.91 | -5.727 | -872.95 | 29.285 | -8.6380 | 0.4422 | 9.0803 | 0.2323 | -0.4066 | 0.6389 | 2.855 | 143.21 | 3.637 | 497.70 | 839.90 | -9.37 |
| 17 | **DP-6,7-ADTN (AG)** | -117.91 | -5.727 | -864.98 | 29.285 | -8.4451 | 0.5004 | 8.9455 | 0.2320 | -0.4061 | 0.6380 | 2.154 | 144.55 | 3.577 | 502.90 | 843.24 | -10.03 |
| 18 | **cirazoline (AG)** | -95.31 | -4.395 | -613.39 | 25.265 | -8.8946 | 0.0296 | 8.9241 | 0.2598 | -0.5042 | 0.7639 | 3.884 | 121.32 | 3.892 | 420.86 | 686.31 | -3.11 |
| 19 | **St-587 (AG)** | -141.86 | -4.526 | -724.37 | -10.308 | -8.8692 | -0.3630 | 8.5062 | 0.5075 | -0.4968 | 1.0043 | 6.154 | 121.17 | 3.986 | 420.73 | 655.04 | -7.34 |
| 20 | **M-7 (AG)** | -95.25 | -4.176 | -599.81 | 23.753 | -8.6376 | 0.4473 | 9.0849 | 0.2324 | -0.3926 | 0.6250 | 2.929 | 112.76 | 3.636 | 397.22 | 644.20 | -10.42 |
| 21 | **guanabenz (AG)** | -100.03 | -4.048 | -489.01 | -10.685 | -8.7619 | -0.7600 | 8.0019 | 0.4870 | -0.5359 | 1.0230 | 3.476 | 126.20 | 3.947 | 392.95 | 617.63 | -15.32 |
| 22 | **oxymetazoline (AG)** | -113.44 | -5.858 | -839.33 | 25.783 | -8.8068 | 0.4420 | 9.2489 | 0.2485 | -0.5047 | 0.7532 | 3.625 | 146.32 | 3.691 | 495.16 | 834.40 | -2.88 |
| 23 | **prazosin (AN)** | -183.60 | -6.791 | -1397.45 | 38.292 | -8.4524 | -0.4241 | 8.0283 | 0.4583 | -0.4739 | 0.9322 | 2.742 | 228.46 | 4.037 | 626.61 | 1062.40 | -10.18 |
| 24 | **phentoloamine (AN)** | -123.07 | -5.706 | -880.32 | 30.960 | -8.2401 | 0.1822 | 8.4223 | 0.2635 | -0.4786 | 0.7421 | 1.262 | 174.67 | 4.131 | 521.37 | 864.88 | -9.79 |
| 25 | **dihydroergotamine (AN)** | -268.33 | -11.040 | -2913.76 | 65.157 | -8.3384 | 0.1201 | 8.4585 | 0.3946 | -0.5330 | 0.9277 | 5.736 | 309.95 | 4.400 | 763.30 | 1467.94 | -9.00 |
| 26 | **clozapine (AN)** | -137.85 | -6.538 | -1025.71 | 19.279 | -8.4831 | -0.4848 | 7.9982 | 0.3391 | -0.3906 | 0.7297 | 4.373 | 200.14 | 3.946 | 541.05 | 917.36 | -3.97 |
| 27 | **corynanthine (AN)** | -160.84 | -6.986 | -1335.93 | 41.814 | -8.1099 | 0.3790 | 8.4889 | 0.3463 | -0.3871 | 0.7334 | 2.701 | 193.75 | 3.784 | 577.67 | 996.58 | -6.35 |
| 28 | **azapetine (AN)** | -94.99 | -4.951 | -680.90 | 34.577 | -8.8360 | -0.0080 | 8.8279 | 0.1070 | -0.3884 | 0.4955 | 1.033 | 150.92 | 4.365 | 434.72 | 735.49 | -2.01 |
| 29 | **yohimbine (AN)** | -160.84 | -6.988 | -1322.15 | 41.751 | -8.0486 | 0.4395 | 8.4881 | 0.3558 | -0.3899 | 0.7457 | 3.842 | 195.42 | 3.781 | 582.11 | 1004.96 | -5.55 |
| 30 | **piperoxane (AN)** | -105.49 | -4.704 | -703.13 | 28.728 | -8.9110 | 0.4144 | 9.3253 | 0.1201 | -0.4029 | 0.5230 | 1.270 | 128.10 | 3.747 | 453.60 | 727.47 | -2.72 |
| 31 | **tolazoline (AN)** | -67.79 | -3.488 | -371.17 | 16.113 | -8.8552 | 0.2393 | 9.0946 | 0.2555 | -0.4976 | 0.7531 | 2.962 | 92.61 | 3.910 | 364.08 | 558.59 | -3.37 |
| 32 | **mianserine (AN)** | -111.43 | -5.681 | -840.61 | 29.007 | -8.7419 | 0.2200 | 8.9619 | 0.2398 | -0.3983 | 0.6381 | 1.827 | 160.74 | 4.356 | 470.67 | 795.40 | -1.31 |
| 33 | **rauwolscine (AN)** | -160.83 | -6.975 | -1381.16 | 42.127 | -8.1371 | 0.3577 | 8.4948 | 0.3723 | -0.3783 | 0.7506 | 2.507 | 191.09 | 3.786 | 545.07 | 963.30 | -5.97 |

**Table 4S.** The numerical values of 16 structural parameters derived from the quantum-chemical calculations in the aquatic environment for all 33 considered compounds.

| **No.** | **Compound** | **TE** | **BE** | **EE** | **HF** | **E_HOMO** | **E_LUMO** | **EG** | **MAX_POS** | **MAX_NEG** | **DELTA_Q** | **TDM** | **MPOL** | **EL** | **SA** | **V** | **HE** |
| --- | --- | --- | --- | --- | --- | --- | --- | --- | --- | --- | --- | --- | --- | --- | --- | --- | --- |
| 1 | **lofexidine (AG)** | -112.24 | -4.521 | -659.76 | 0.510 | -9.0112 | -0.3260 | 8.6851 | 0.2639 | -0.5055 | 0.7694 | 4.090 | 116.76 | 3.848 | 416.99 | 688.35 | -2.44 |
| 2 | **clonidine (AG)** | -97.51 | -4.111 | -505.99 | -7.611 | -8.6380 | -0.4820 | 8.1560 | 0.4355 | -0.5120 | 0.9476 | 4.099 | 114.00 | 3.799 | 388.35 | 616.10 | -5.14 |
| 3 | **naphazoline (AG)** | -87.29 | -4.392 | -551.90 | 25.653 | -8.7844 | -0.4151 | 8.3693 | 0.2598 | -0.5011 | 0.7608 | 3.076 | 132.83 | 3.682 | 424.12 | 674.57 | -3.56 |
| 4 | **tiamenidine (AG)** | -86.24 | -3.491 | -455.78 | 5.722 | -8.4287 | 0.0650 | 8.4937 | 0.4961 | -0.4862 | 0.9823 | 3.799 | 108.50 | 3.522 | 382.35 | 603.23 | -5.63 |
| 5 | **xylazine (AG)** | -86.07 | -4.164 | -552.31 | 24.440 | -8.6412 | 0.3019 | 8.9437 | 0.2687 | -0.4169 | 0.6856 | 1.771 | 132.28 | 3.665 | 420.79 | 687.77 | -1.79 |
| 6 | **tramazoline (AG)** | -91.98 | -4.822 | -598.65 | 18.222 | -8.5056 | 0.2454 | 8.7510 | 0.4133 | -0.4887 | 0.9020 | 3.216 | 130.91 | 3.937 | 433.18 | 697.11 | -3.49 |
| 7 | **xylometazoline (AG)** | -101.81 | -5.844 | -751.81 | 23.574 | -8.7313 | 0.2829 | 9.0141 | 0.2616 | -0.5000 | 0.7615 | 3.072 | 142.91 | 3.794 | 493.50 | 819.40 | 0.76 |
| 8 | **tetryzoline (AG)** | -83.75 | -4.451 | -541.20 | 21.157 | -9.0255 | 0.5890 | 9.6144 | 0.2331 | -0.4643 | 0.6974 | 2.366 | 116.15 | 3.888 | 414.02 | 657.92 | -2.00 |
| 9 | **methoxamine(AG)** | -102.24 | -3.991 | -622.78 | 23.975 | -8.7961 | 0.3569 | 9.1529 | 0.1925 | -0.4775 | 0.6700 | 1.321 | 109.32 | 3.741 | 419.99 | 671.52 | -6.87 |
| 10 | **phenylephrine (AG)** | -79.29 | -3.217 | -410.00 | 18.599 | -9.7961 | 0.2450 | 9.3569 | 0.2201 | -0.4502 | 0.6703 | 0.531 | 88.55 | 3.764 | 371.87 | 568.47 | -13.84 |
| 11 | **DPI (AG)** | -93.63 | -3.512 | -495.69 | 15.855 | -8.1717 | 0.3982 | 8.5698 | 0.4743 | -0.5064 | 0.9807 | 4.995 | 115.61 | 3.688 | 387.07 | 592.65 | -21.16 |
| 12 | **amidephrine (AG)** | -111.75 | -3.871 | -669.59 | 25.339 | -9.3226 | -0.9255 | 8.3971 | 2.9637 | -0.9884 | 3.9522 | 5.429 | 116.39 | 3.823 | 446.73 | 715.62 | -11.84 |
| 13 | **Sgd 101/75(AG)** | -95.96 | -4.481 | -588.20 | 13.463 | -7.7370 | 0.0742 | 7.8112 | 0.4587 | -0.4693 | 0.9280 | 6.813 | 139.01 | 3.659 | 423.01 | 671.15 | -10.37 |
| 14 | **DP-5-ADTN (AG)** | -106.27 | -5.701 | -794.00 | 27.408 | -8.8452 | 0.6012 | 9.4464 | 0.2169 | -0.4017 | 0.6185 | 2.670 | 138.64 | 3.774 | 486.88 | 815.80 | -3.69 |
| 15 | **DP-7-ADTN (AG)** | -106.27 | -5.702 | -785.67 | 27.368 | -8.5731 | 0.6182 | 9.1914 | 0.2171 | -0.3900 | 0.6071 | 1.323 | 140.22 | 3.719 | 491.04 | 818.43 | -4.48 |
| 16 | **DP-5,6-ADTN (AG)** | -117.91 | -5.726 | -869.58 | 29.302 | -8.6488 | 0.4440 | 9.0927 | 0.2263 | -0.3910 | 0.6173 | 3.252 | 144.52 | 3.654 | 500.29 | 838.57 | -9.27 |
| 17 | **DP-6,7-ADTN (AG)** | -117.91 | -5.726 | -867.76 | 29.309 | -8.6229 | 0.4069 | 9.0298 | 0.2239 | -0.4006 | 0.6245 | 2.818 | 144.72 | 3.629 | 500.74 | 842.20 | -10.16 |
| 18 | **cirazoline (AG)** | -95.31 | -4.395 | -611.35 | 25.287 | -8.9320 | 0.1492 | 9.0812 | 0.2533 | -0.4892 | 0.7425 | 4.102 | 122.13 | 3.893 | 421.70 | 689.21 | -2.78 |
| 19 | **St-587 (AG)** | -141.86 | -4.525 | -725.18 | -10.286 | -8.9682 | -0.3997 | 8.5685 | 0.5072 | -0.4816 | 0.9889 | 6.136 | 120.47 | 3.976 | 415.49 | 656.89 | -7.31 |
| 20 | **M-7 (AG)** | -95.25 | -4.175 | -601.40 | 23.799 | -8.6562 | 0.4427 | 9.0990 | 0.2260 | -0.3847 | 0.6107 | 3.094 | 112.56 | 3.648 | 394.28 | 644.16 | -10.23 |
| 21 | **guanabenz (AG)** | -100.03 | -4.049 | -489.31 | -10.712 | -8.9733 | -0.6793 | 8.2940 | 0.4789 | -0.5286 | 1.0074 | 3.525 | 121.96 | 4.145 | 395.09 | 622.41 | -15.39 |
| 22 | **oxymetazoline (AG)** | -113.44 | -5.858 | -838.92 | 25.766 | -8.7515 | 0.4512 | 9.2027 | 0.2561 | -0.5145 | 0.7706 | 3.945 | 146.66 | 3.701 | 496.41 | 834.66 | -3.07 |
| 23 | **prazosin (AN)** | -183.60 | -6.789 | -1401.16 | 38.337 | -8.4872 | -0.4649 | 8.0224 | 0.4533 | -0.4764 | 0.9296 | 2.893 | 227.54 | 4.047 | 629.63 | 1062.03 | -10.06 |
| 24 | **phentoloamine (AN)** | -123.07 | -5.703 | -881.26 | 31.003 | -8.1693 | 0.1586 | 8.3279 | 0.2720 | -0.4917 | 0.7637 | 1.844 | 175.91 | 4.054 | 519.60 | 867.48 | -9.54 |
| 25 | **dihydroergotamine (AN)** | -268.33 | -11.038 | -2915.93 | 65.202 | -8.3389 | 0.1813 | 8.5202 | 0.3867 | -0.5263 | 0.9130 | 4.839 | 309.21 | 4.386 | 760.92 | 1465.66 | -8.92 |
| 26 | **clozapine (AN)** | -137.85 | -6.537 | -1025.94 | 19.294 | -8.5109 | -0.4664 | 8.0445 | 0.3467 | -0.4019 | 0.7486 | 4.502 | 200.27 | 3.579 | 540.34 | 917.97 | -3.98 |
| 27 | **corynanthine (AN)** | -160.84 | -6.988 | -1335.20 | 41.762 | -8.1772 | 0.3121 | 8.4892 | 0.3493 | -0.3800 | 0.7293 | 1.831 | 193.45 | 3.786 | 574.90 | 993.95 | -7.57 |
| 28 | **azapetine (AN)** | -94.99 | -4.950 | -682.39 | 34.586 | -8.8461 | -0.0044 | 8.8525 | 0.1072 | -0.3837 | 0.4910 | 1.045 | 150.39 | 4.370 | 431.98 | 732.69 | -1.98 |
| 29 | **yohimbine (AN)** | -160.84 | -6.986 | -1322.20 | 41.806 | -8.0236 | 0.4502 | 8.4737 | 0.3439 | -0.3795 | 0.7234 | 4.023 | 195.67 | 3.773 | 584.81 | 1008.16 | -5.67 |
| 30 | **piperoxane (AN)** | -105.49 | -4.704 | -702.78 | 28.733 | -8.9119 | 0.4174 | 9.3293 | 0.1201 | -0.3955 | 0.5156 | 1.351 | 128.37 | 3.744 | 450.24 | 728.66 | -2.71 |
| 31 | **tolazoline (AN)** | -67.79 | -3.487 | -370.78 | 16.132 | -8.7420 | 0.2873 | 9.0293 | 0.2626 | -0.5059 | 0.7685 | 3.082 | 92.88 | 3.911 | 364.87 | 558.58 | -3.20 |
| 32 | **mianserine (AN)** | -111.43 | -5.681 | -840.83 | 29.012 | -8.7920 | 0.2097 | 9.0016 | 0.2337 | -0.3941 | 0.6278 | 1.930 | 160.75 | 4.390 | 469.24 | 795.24 | -1.30 |
| 33 | **rauwolscine (AN)** | -160.83 | -6.975 | -1386.30 | 42.119 | -8.1304 | 0.3511 | 8.4815 | 0.3754 | -0.3859 | 0.7613 | 2.241 | 191.76 | 3.781 | 539.93 | 958.06 | -5.94 |

**Table 5S.** The numerical values of the 10 structural parameters derived from quantum-chemical calculations *in vacuo* for 22 considered compounds (*α*-adrenergic agonists) obtained by the PCM (Polarizable Continuum Model) method.

| **Compound** | **TE** | **ESE** | **E_HOMO** | **E_LUMO** | **EG** | **MAX_POS** | **MAX_NEG** | **DELTA_Q** | **TDM** | **IPOL** |
| --- | --- | --- | --- | --- | --- | --- | --- | --- | --- | --- |
| **lofexidine** | -1526.25 | 4141.92 | -9.0365 | 3.1440 | 12.1805 | 0.5561 | -0.7008 | 1.2569 | 4.6000 | 135.26 |
| **clonidine** | -1428.37 | 3422.06 | -8.8264 | 2.9162 | 11.7426 | 0.8431 | -0.7635 | 1.6066 | 4.1449 | 119.85 |
| **naphazoline** | -647.24 | 3785.66 | -7.9149 | 2.5494 | 10.4643 | 0.5631 | -0.7022 | 1.2652 | 2.8672 | 144.54 |
| **tiamenidine** | -1329.10 | 3305.15 | -8.6226 | 3.5453 | 12.1679 | 0.8359 | -0.8095 | 1.6455 | 3.7116 | 116.13 |
| **xylazine** | -970.17 | 3913.27 | -8.4436 | 3.9940 | 12.4376 | 0.3994 | -0.7798 | 1.1792 | 1.8445 | 142.58 |
| **tramazoline** | -665.57 | 4376.64 | -8.0604 | 3.8033 | 11.8637 | 0.8516 | -0.8397 | 1.6913 | 2.9655 | 142.84 |
| **xylometazoline** | -728.81 | 5844.99 | -8.5023 | 3.7992 | 12.3015 | 0.5573 | -0.6983 | 1.2556 | 2.6956 | 169.11 |
| **tetryzoline** | -610.53 | 3272.46 | -8.5576 | 3.8705 | 12.4281 | 0.5533 | -0.7006 | 1.2539 | 2.2586 | 131.42 |
| **methoxamine** | -705.47 | 3603.87 | -8.0466 | 3.7638 | 11.8104 | 0.4137 | -0.7295 | 1.1432 | 2.6150 | 122.30 |
| **phenylephrine** | -552.57 | 2799.91 | -8.4281 | 3.7225 | 12.1506 | 0.3989 | -0.6640 | 1.0629 | 0.7728 | 98.20 |
| **DPI** | -660.30 | 3598.68 | -7.2474 | 4.3353 | 11.5827 | 0.8776 | -0.7331 | 1.6108 | 5.4099 | 111.33 |
| **amidephrine** | -1118.94 | 5284.93 | -8.6482 | 3.5551 | 12.2033 | 1.5931 | -0.8796 | 2.4727 | 5.9658 | 131.22 |
| **Sgd 101/75** | -696.36 | 4194.82 | -6.9774 | 3.4310 | 10.4084 | 0.8868 | -0.7364 | 1.6232 | 5.6535 | 139.05 |
| **DP-5-ADTN** | -749.79 | 5235.37 | -8.0915 | 4.1295 | 12.2210 | 0.3683 | -0.6678 | 1.0361 | 2.4609 | 162.82 |
| **DP-7-ADTN** | -749.79 | 5581.69 | -7.9483 | 3.9453 | 11.8936 | 0.3857 | -0.6606 | 1.0464 | 0.9249 | 163.92 |
| **DP-5,6-ADTN** | -824.65 | 5992.83 | -7.8871 | 4.0893 | 11.9764 | 0.3769 | -0.7046 | 1.0815 | 3.1815 | 166.37 |
| **DP-6,7-ADTN** | -824.65 | 6327.43 | -7.7241 | 4.0430 | 11.7671 | 0.3683 | -0.6949 | 1.0632 | 2.3728 | 167.37 |
| **cirazoline** | -685.43 | 4459.41 | -8.6474 | 3.8324 | 12.4798 | 0.5122 | -0.7191 | 1.2313 | 1.6699 | 135.72 |
| **St-587** | -1305.11 | 5257.78 | -8.6283 | 3.1924 | 11.8207 | 1.1728 | -0.7305 | 1.9033 | 5.2076 | 122.93 |
| **M-7** | -668.50 | 3399.20 | -7.8727 | 4.1203 | 11.9930 | 0.3775 | -0.7050 | 1.0825 | 3.3652 | 125.51 |
| **guanabenz** | -1444.33 | 4263.11 | -8.3859 | 2.0901 | 10.4760 | 0.8605 | -0.8041 | 1.6645 | 4.0580 | 130.70 |
| **oxymetazoline** | -803.66 | 6416.99 | -7.7864 | 4.1257 | 11.9121 | 0.5507 | -0.6965 | 1.2472 | 3.9581 | 172.00 |
